# The Epidemiology of Snakebite in Bhutan: A Retrospective Study

**DOI:** 10.1002/puh2.70077

**Published:** 2025-07-04

**Authors:** Tshokey Tshokey, Rixin Jamtsho, Sangay Rinchen

**Affiliations:** ^1^ Department of Pathology and Laboratory Medicine Jigme Dorji Wangchuck National Referral Hospital (JDWNRH) Thimphu Bhutan; ^2^ Faculty of Postgraduate Medicine Khesar Gyalpo University of Medical Sciences of Bhutan (KGUMSB) Thimphu Bhutan; ^3^ Medical Education Centre for Research Innovation and Technology (MECRIT) Khesar Gyalpo University of Medical Sciences of Bhutan Thimphu Bhutan; ^4^ National Centre for Animal Health, Serbithang Thimphu Bhutan

**Keywords:** Bhutan | epidemiology | neglected tropical diseases | snakebite

## Abstract

Bhutan is a tropical country where snakebite is supposedly common, but official data are scanty and unmethodical. Deaths from snakebites were reported from areas where snakebites are common. Four‐year (2018–2021) data of snakebite from 45 Bhutanese hospitals were collected and analysed to describe the burden and map by districts. A total of 371 snakebites were recorded from 45 hospitals during the 4 years. Most cases were seen in the southern and central parts of the country. There was a definite rise in the number of cases in the warmer months, starting from March and peaking between June and August. About 240 (65%) of the bites occurred in males, and the highest number of snakebites occurred during farming (*n* = 100, 27%), bush walking (*n* = 42, 11.3%), herding (*n* = 15, 4%) and trekking (*n* = 1, 0.2%). The most common anatomical bite site was the leg (*n* = 167, 45.01%), followed by the hand (*n* = 81, 21.8%), finger (*n* = 56, 15.09%), toes (*n* = 11, 2.96%), thigh region (*n* = 6, 1.6%), head and face (*n* = 3, 0.8%), chest and shoulder (*n* = 3, 0.8%) and abdomen (*n* = 1, 0.3%). Most snakes were unidentified (*n* = 266, 71.7%). Those identified were vipers (*n* = 74, 19.9%), rat snakes (*n* = 12, 3.2%), kraits (*n* = 7, 1.88%), cobras (*n* = 6, 1.61%), river snakes (*n* = 5, 1.34%) and wolf snakes (*n* = 1, 0.26%). Forty‐three (11.6%) of the bite victims had resorted to non‐medical home treatment. Even with an obvious underreporting, snakebite is a significant public health problem, and Bhutan should embrace more public health and clinical activities to prevent morbidities and mortalities from snakebite.

## Graphical abstract

This study reports 371 cases of snakebites in 4 years (2018–2021) from 45 Bhutanese hospitals. Snakebite is a neglected public health problem with no consistent recording and reporting system, no national treatment guidelines and no public health program activities in Bhutan.

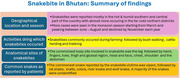

## 1 Introduction

Bhutan is a tropical country with diverse climatic conditions. Snakebites are supposedly common in the hot southern belt and the warm central belt of the country. It is not uncommon to hear or read about deaths from snakebites among rural people or in national media. The global statistics on snakebites in 2014 reported that countries in South Asia have the highest snakebite rates, and within South Asia, Bhutan has the highest snakebite rate per 1000 population, at 4.13 bites, followed by Nepal at 2.89 bites [1]. Globally, more than 5.8 billion people are at risk of snakebite envenoming, and snakebites kill between 81,000 and 138,000 people annually [2, 3]. Locally, there are several reports of deaths due to snakebites in different districts, especially in the south and the central parts of the country [4, 5, 6]. A study in the southern district of Sarpang recorded 78 snakebite cases treated in the Central Regional Referral Hospital over a period of 3 years (2013–2015). Of the total, 28 (36%) cases developed signs and symptoms of envenomation, and the remaining 50 (64%) were found to be cases of non‐venomous bites. Snakebites occurred more in males than females and dominantly occurred in the young and active age groups of 21–50 years. Most of the venomous bites (68%) occurred during the monsoon season, particularly between the months of May and August of the year [7]. Another study revealed poor knowledge of health workers on snakebites. About 63% of the victims visited local (traditional) healers prior to seeking medical help from hospitals. About 83% of the snakebite victims also used a tight tourniquet as a first aid in snakebite management [8]. The 2022 Annual Health Bulletin (AHB) of the Ministry of Health (MoH) reported 186, 206, 220 and 203 snakebite cases in the years 2018, 2019, 2020 and 2021, respectively, in Bhutan [9]. Despite this high number, at the country level, Bhutan has poor data on snakebites, which are collected passively, and currently, snakebite is not a public health priority.

In 2017, the World Health Assembly adopted a resolution on snakebite envenomation and categorized snakebite as a neglected tropical disease. This prompted the WHO to develop comprehensive strategies on snakebite prevention and preservation [10]. In Bhutan's health system, snakebite is a seriously neglected tropical disease, and this inference is drawn from several factors such as (a) snakebite is not a notifiable disease [11], (b) there is no registry of snakebites, (c) there is no formal curriculum or continued education on snakebite, (d) no national guidelines on management of snakebites, (e) no strategy in place to deploy snakebite‐trained health professionals to high‐risk areas and (f) no strategic supply of anti‐snake venoms to health centres in high‐snakebite burden areas. All these neglects possibly lead to inadequate management of snakebites and cause preventable deaths in the country.

Thus, the need for good data on snakebite, the need for awareness and expertise amongst health professionals and the need for all health centres to be snakebite management ready (by manpower, expertise and infrastructure) are genuine. This study was carried out to find out the epidemiology of snakebites and reveal the gaps.

## 2 Materials and Methods

### 2.1 Study Design

This was a descriptive observational study involving the collection and analysis of data on snakebites recorded in the past 4 years in the Bhutanese hospitals.

### 2.2 Data Source and Study Period

Data were collected from hospital records of snakebites in any form, including inpatient records, admission registers, individual patient files archived in hospital stores and online/offline spreadsheets maintained by the respective hospitals. The study included data for 4 years (2018–2021).

### 2.3 Sample Size and Study Sites

There was no predefined sample size for this study. The study enrolled the entire list of snakebite cases available in Bhutan's 45 hospitals, including one national referral hospital, two regional referral hospitals and the remaining consisting of district hospitals and primary health centres (10‐bedded basic hospitals). There were no exclusion criteria, and cases with incomplete information were also included in the analysis.

### 2.4 Questionnaire Development and Administration

A study questionnaire was developed specifically for this study with references from snakebite management standards and international literature of similar studies. The questionnaire was field tested and revised before circulating for data collection. The information collected in the questionnaire included demographic details and geographical location, snakebite information (including the month the bite occurred, anatomical site of the bite and activities during the bite), snake identification, non‐medical home treatment provided before visiting health centres and outcomes. The questionnaire was administered using a mobile phone–based application, EpiCollect5 software [12]. Trained health workers (doctors, nurses and allied health staff) collected and entered the data individually from their respective hospitals.

### 2.5 Data Analysis

The data collected in Epicollect5 software was extracted into Microsoft Excel and analysed using R statistical package [13]. The analysis was descriptive of numbers, incidence/prevalence of snakebites, the pattern over years, the demographic and risk pattern of snakebite victims and the geographical mapping of the snakebite. Associations among different variables of interest were assessed using student's *t*‐test, Pearson's chi‐squared test and Fisher's exact test based on relevance. During the analysis, the missing data and levels of a variable such as ‘Others’ and ‘not documented’ were dropped. For analysis, the 20 districts of the country were divided into ‘warm’ and ‘cold’ districts. Districts, such as Chhukha, Dagana, Pema Gatshel, Punakha, Samdrup Jongkhar, Samtse, Sarpang, Wangdue Phodrang, Zhemgang, Lhuntse, Trashigang, Mongar and Tsirang, were categorized as warm districts, whereas the remaining were categorized as cold.

## 3 Results

**FIGURE 1:**
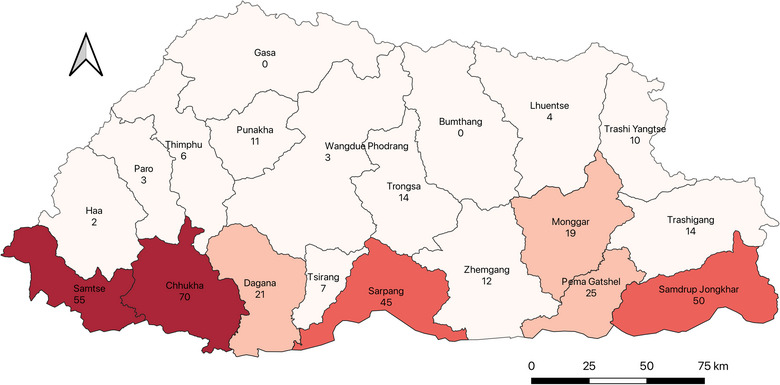
Distribution of snakebites in Bhutan by numbers from 2018 to 2021.

Data from 45 hospitals in Bhutan's 20 districts were included in the study. Record keeping and archiving varied highly among hospitals, ranging from maintenance of registers specific to snakebites, archiving the whole of individual patient files in hard copy and maintaining online or offline spreadsheets in emergency units, injection rooms and wards. Between 2018 and 2021, a total of 371 cases of snakebites were recorded in these hospitals. The southern districts with a warm‐to‐hot sub‐tropical climate saw the highest snakebite cases, and the number of cases decreased as we moved higher towards the north, with two districts in the far north (Bumthang and Gasa) having no snakebite cases, as shown in Table 1 and Figure 1. The number of snakebites was significantly higher in the warm districts compared to the cold districts during the 4‐year period (*p* value = 0.006).

**TABLE 1:** Yearly snakebites by district in 4 years (2018–2021).

Sl. no.	Districts (no. of hospitals)	Year				
		2018	2019	2020	2021	Total
1	Bumthang (1)	0	0	0	0	0
2	Chhukha (5)	0	26	19	25	70
3	Dagana (3)	0	6	3	12	21
4	Gasa (1)	0	0	0	0	0
5	Haa (1)	0	2	0	0	2
6	Lhuentse (1)	0	1	0	3	4
7	Mongar (2)	0	6	6	7	19
8	Paro (1)	0	1	2	0	3
9	Pemagatshel (2)	0	7	9	9	25
10	Punakha (1)	0	4	4	3	11
11	Samdrup Jongkhar (5)	0	3	16	31	50
12	Samtse (4)	0	19	23	13	55
13	Sarpang (3)	0	18	15	12	45
14	Thimphu (2)	0	3	1	2	6
15	Trashi Yangtse (2)	0	4	2	4	10
16	Trashigang (7)	2	4	3	5	14
17	Trongsa (2)	0	1	8	5	14
18	Tsirang (2)	0	0	4	3	7
19	Wangdue Phodrang (2)	0	1	1	1	3
20	Zhemgang (4)	0	6	6	0	12
	**Total**	**2**	**112**	**122**	**135**	**371**

**FIGURE 2:**
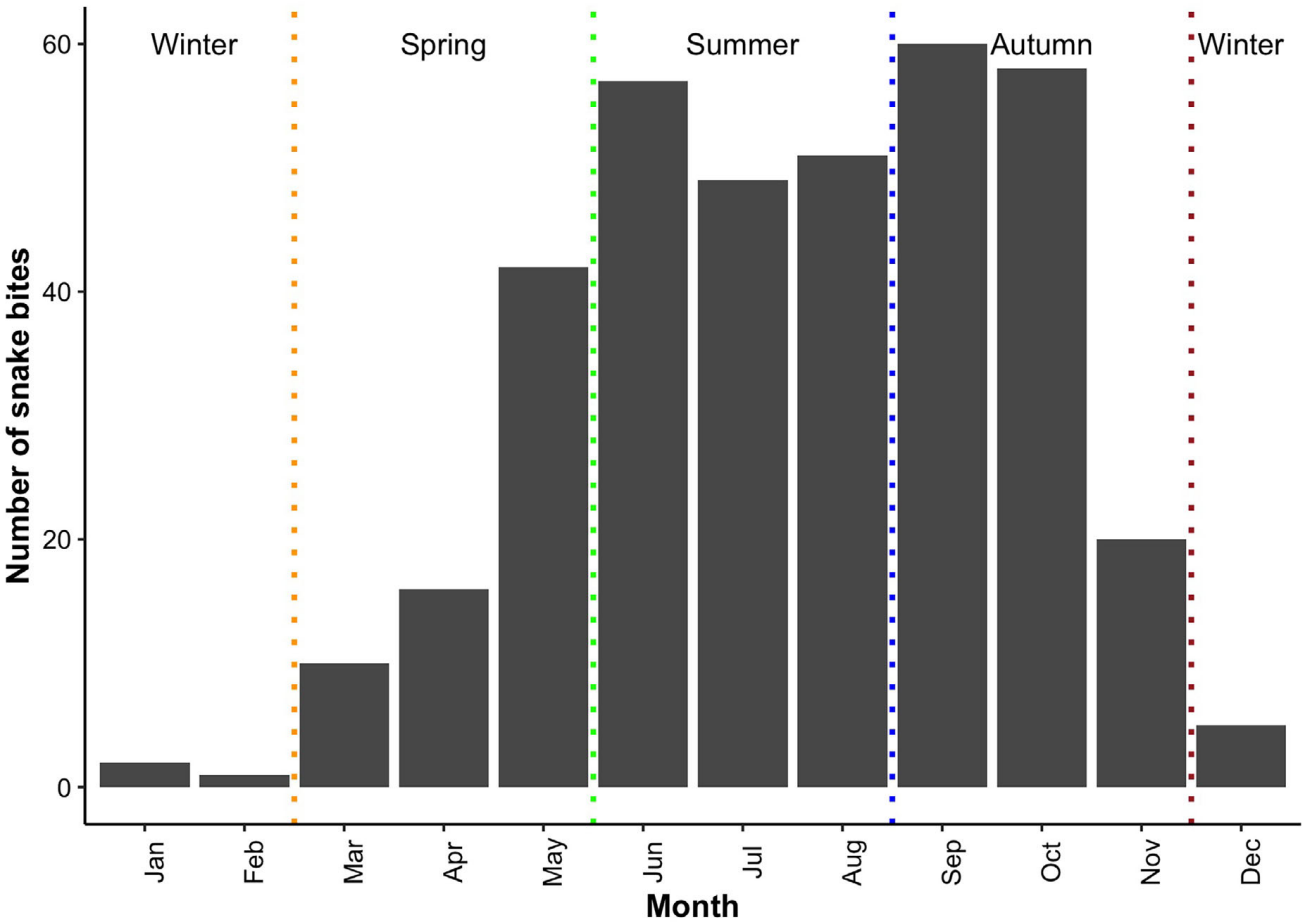
Monthly and seasonal pattern of snakebite cases in Bhutan.

The number of snakebites revealed a definite rise in the hot summer and monsoon season, starting from March and peaking between June and August and declined by November. The highest number of bites (*n* = 157, 42.3%) were reported during the summer season, followed by autumn (*n* = 138, 37.2%). There were only few cases in the cold winter season from December to February (Figure 2). Only eight (2.1%) snakebites were reported during the winter season. Of these, two were bitten by a krait, two by a viper, whereas four were not documented.

Of the 371 snakebite victims, 240 (65%) were males and 131 (35%) were females. The mean age of the bite victims was 38.7 years (SD = 16.8). The youngest bite victim was a 1‐year‐old girl and she was bitten while playing in a playground. The oldest victim was a 93‐year‐old lady but the activity during which the bite occured was not mentioned in the medical record. Among the activities listed in the study questionnaire, the highest number of snakebites occurred during the farming activities (*n* = 102, 27.49%), followed by bush walking (*n* = 42, 11.3%), herding (*n* = 15, 4%) and trekking (*n* = 1, 0.2%). Of the total cases, 213 (57%) were reported as others and 142 (38.3%) cases did not have documentation on activities engaged in during the bite. Of the rest (*n* = 69), 27 (39.13%) reported being bitten while working at home or in the home premises, 16 (4.3%) while walking, 11 (15%) while visiting toilet, 5 (7.24%) while playing, 5 (7.24%) while undertaking activities by the river side, 3 (4.34%) while engaging in activities in the forest and 2 (2.89%) while handling or playing with snakes.

**FIGURE 3:**
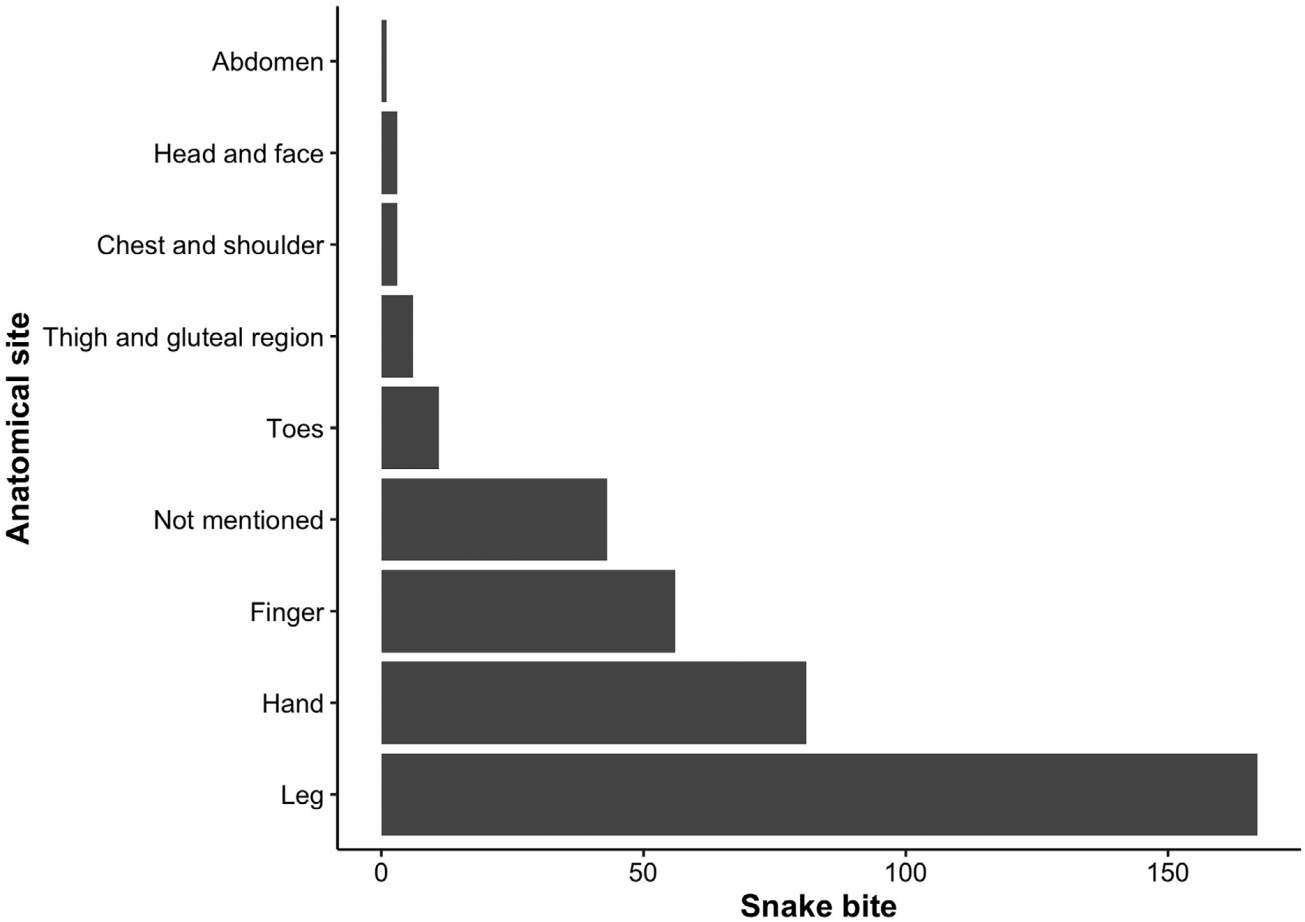
Common anatomical sites of snakebite.

The most common anatomical site of a bite was leg (*n* = 167, 45.01%), followed by hand (*n* = 81, 21.83%), finger (*n* = 56, 15.09%), toes (*n* = 11, 2.96%), thigh and gluteal region (*n* = 6, 1.61%), head and face (*n* = 3, 0.80%), chest and shoulder (*n* = 3, 0.08%) and abdomen (*n* = 1, 0.27%). The anatomical site of the bite was not recorded for 43 (*n* = 11.6%) cases (Figure 3.)

There is a significant association between the activities that bite victims were engaged in and the anatomical site of snakebites (Fisher test, *p* value = 0.002). It was found that most snakebites happened when people were involved in farming and bushwalking. When the bite occurred during farming, the commonest site was the hand, followed by legs, and when it happened during bushwalking, the commonest site was legs, followed by the hands (Table 2).

**TABLE 2:** Relation between activities and anatomical sites of snakebite.

	Anatomical site of snakebite					
Activities involved	Finger	Hand	Leg	Thigh/Gluteal region	Toes	Total
Bush walking	3	6	27	1	2	**39**
Farming	15	39	34	2	3	**93**
Herding	1	2	12	0	0	**15**
Trekking	0	0	1	0	0	**1**
**Total**	**19**	**47**	**74**	**3**	**5**	**148**

*Note:* Fisher test *p* value = 0.009.

In this study, vipers were the commonest snakes responsible for snakebites in the country. From a total of 371 snakebites, 74 (19.9%) were reported to be due to vipers, followed by rat snakes (*n* = 12, 3.2%), kraits (*n* = 7, 1.88%), cobras (*n* = 6, 1.61%), river snakes (*n* = 5, 1.34%) and wolf snakes (*n* = 1, 0.26%). However, in 266 of the 371 cases (71.69%), the snake was not identified (Table 3).

**TABLE 3:** Types of snakes identified by the bite victims.

Year	Cobra	Kraits	Rat snake	River snake	Viper	Wolf snake	Unknown	Total
2018	0	0	0	0	0	0	2	2
2019	0	4	3	1	22	0	82	112
2020	2	1	7	3	17	1	91	122
2021	4	2	2	1	35	0	91	132
**Total**	**6**	**7**	**12**	**5**	**74**	**1**	**266**	**371**

Forty‐three (11.6%) of the bite victims reported resorting to non‐medical home treatment before availing medical services. The most common home treatment practices followed were cutting the bite area and sucking the blood, application of herbal preparation and application of tight tourniquet.

## 4 Discussion

This study reports enough data and evidence to show that snakebite is common in Bhutan, although it remains a neglected public health problem. The website [1] which reported Bhutan as having the highest snakebite rate per 1000 population was discontinued since 2015, and the data source is questionable, although it is still available in public domain. This outdated and probably irrelevant data showing a high rate for Bhutan could be due to the smaller denominator resulting from the small population of the country. The AHB of the MoH reported 186, 206, 220 and 203 snakebite cases in the years 2018, 2019, 2020 and 2021, respectively, in Bhutan [9]. During the corresponding years, only three deaths were reported in 2019, with no deaths in 2018, 2020 and 2021. This annual figure is derived from passive reports submitted by the health centres, and the reported figures may be a gross underestimate of the actual cases because reporting is not methodical and it is not mandatory. The observation of non‐uniform, inconsistent and highly variable record‐keeping methods in different hospitals, which were probably dependent on the proactiveness of the hospital staff, could have resulted in discrepancies of annual data that is reported to the MoH and ultimately published in the AHB. In contrast to these annual reports submitted to the MoH by district health sectors, the present study recorded only 371 snakebites in 4 years from 45 hospitals. This is a gross underestimate of national snakebite data and warrants proper institution of snakebite registry by the MoH. Due to the lack of proper registry, the morbidity and mortality probably go unnoticed in the eyes of the policy makers. The record of only two snakebites in 2018 compared to the next years clearly reveals that as the years pass, the hospital records are not maintained or archived properly. Instituting a mandatory notification of snakebites and deaths associated with it may be able to capture true data.

The incidence of snakebites was significantly high during farming and bushwalking, most commonly involving the hands and the legs, respectively. These warrants creating awareness amongst farmers and bush walkers on the snakebite prevalent areas and the means to prevent snakebites. It may also help in making the public aware of the basic first aid practices at the time of bite until arrival to the health facilities. All these require concerted efforts from the MoH through public awareness and clinical guidelines.

In this study, vipers were identified as the most common snakes responsible for the bites. These identifications were based on reports by snakebite victims as seen during the bites or dead bodies of killed snakes or photographs taken by victims produced to health workers who are not experts in snake identification. Thus, the reliability of these identifications may be questionable. Nevertheless, it presents an approximation of the prevalent snakes that were involved in the bites. Such information may be useful to make decisions on framing public awareness materials and clinical management guidelines.

Although numerically small (about 12%), the pre‐hospital home treatment of snakebite is a risky practice. The sucking of blood from the bite area may result in infection of the bite areas as well as cause theoretical envenomation of the treatment provider. The application of herbal medicines or local remedies can also cause infection of the bite marks, resulting in complications. Application of tight tourniquet is dangerous because it may cause blood supply occlusion and lead to gangrenous periphery. In addition, all these practices may provide false hope of benefit to the victims and their relatives and cause unnecessary delays in seeking medical care, leading to otherwise preventable complications and death. These observations mandate proper public health education and training of health workers to improve communication to the public in their public health awareness sessions. Additionally, it will benefit to provide local leaders and village health workers with basic first aid practices, especially in areas with high snakebite prevalence. The WHO Regional Action Plan for prevention and control of snakebite envenoming in South‐East Asia 2022–2030 aims to accelerate progress to reduce snakebite‐related death and disability by 50% by 2030 [10]. In line with this, Bhutan must take up the regional plan as a national agenda and initiate different activities both on the public health awareness and clinical front to prevent and control morbidity and mortality due to snakebite envenomation.

This study is not free of limitations. First, due to the inconsistent record‐keeping in different hospitals, some records may have been missed, resulting in a lesser number of cases being reported. Second, there were some records with incomplete data, and these might have affected the analysis although this was minimized by performing analysis only for variables with complete information. Additionally, information on the identification of the involved snake could be highly unreliable and should be interpreted with caution.

## 5 Conclusion and Recommendations

Findings from this study, supported by other gathered information, show enough evidence of significant morbidity and mortality from snakebites in Bhutan. Bhutan must recognize snakebite as a public health problem immediately and implement public health and clinical activities on snakebite in line with the WHO regional strategy to prevent morbidity and mortality. Initial activities to start with should include dedicating a responsible program at the ministry level, establishing methodical snakebite records/registries making snakebites a mandatorily reportable health event, creating public awareness, developing clinical protocols and guidelines and training all categories of health workers on snakebite management.

## Author Contributions

**Tshokey Tshokey** conceived and designed the study. **Tshokey Tshokey**, **Rixin Jamtsho** and **Sangay Rinchen** implemented the study and made the analysis and interpretation of the data and wrote the manuscript; All the authors have critically revised the manuscript for intellectual content; All the authors have read and approved the manuscript in its final version.

## Ethics Statement

Administrative clearance for the study was sought from the Policy and Planning Division of the MoH. Ethics approval was sought from the Institutional Review Board (IRB) of the Khesar Gyalpo University of Medical Sciences of Bhutan (KGUMSB) vide approval no. IRB/Approval/PN21‐041/2021‐22/519.

## Conflicts of Interest

The authors declare no conflicts of interest.

## Data Availability

Data are available on requests.
